# The incidence, mutational status, risk classification and referral pattern of gastro-intestinal stromal tumours in the Netherlands: a nationwide pathology registry (PALGA) study

**DOI:** 10.1007/s00428-017-2285-x

**Published:** 2018-01-08

**Authors:** Arie J. Verschoor, J. V. M. G. Bovée, L. I. H. Overbeek, The PALGA group, J. J. T. H. Roelofs, S. H. Sastrowijoto, A. Willig, R. P. Dutrieux, P. H. van Zwam, M. F. Hamel, M. C. H. Hogenes, C. Bertrand, P. C. W. Hogendoorn, H. Gelderblom

**Affiliations:** 1 Department of Medical Oncology, Leiden University Medical Center, Albinusdreef 2, 2333 ZA Leiden, The Netherlands; 2 Department of Pathology, Leiden University Medical Center, Albinusdreef 2, 2333 ZA Leiden, The Netherlands; 3 PALGA, Randhoeve 225A, 3995 GA Houten, The Netherlands

**Keywords:** Gastrointestinal stromal tumours, Epidemiology, Incidence, Mutation, Pathology, Soft tissue neoplasms

## Abstract

Symptomatic gastrointestinal stromal tumours (GIST) are infrequent with an incidence of 12.7 per million inhabitants in the western population. We studied whether the incidence of GIST has further increased between 2003 and 2012 and assessed the frequency of mutations, risk groups, histological subtypes and immunohistochemistry results. From PALGA, the nationwide Dutch Pathology Registry, pathology excerpts from all patients with a GIST or GIST-like tumour between 2003 and 2012 were retrieved to calculate incidence rates. Full pathology reports were retrieved of resections in 2011 and 2012 to study the frequency of mutations, risk groups, histological subtypes and immunohistochemistry results. The incidence of GIST increased to 17.7 per million inhabitants in 2012 with a median age of 67 years. Mutational analysis was performed in 33.9% of patients with a resection between 2011 and 2012 (KIT mutation 67.5%, PDGFRA 16.3%, wild-type 11.4%). The percentage of high risk patients in the different risk classifications varied from 19.9% to 38.0% depending on the used classification. Only 35.9% of patients had diagnosis or revision of pathology diagnosis within three months in a designated GIST referral centre. No increase in proportion of central pathology reviews was found. Proportion of patients with mutational analysis increased over the years. The registered incidence of GIST, 17.7 per million inhabitants in 2012 in the Netherlands, is still rising. Despite incorporation in the ESMO GIST guidelines since 2008 for mutational testing and since 2010 for central review of pathology, both are performed in a minority of patients.

**Electronic supplementary material:**

The online version of this article (10.1007/s00428-017-2285-x) contains supplementary material, which is available to authorized users.

## Introduction

The most common mesenchymal tumours of the gastrointestinal tract are the gastrointestinal stromal tumours (GISTs) [1]. Clinical behaviour is predicted by primary localisation, tumour size, mitotic index and tumour rupture [2]. The differential diagnosis contains gastrointestinal leiomyoma and leiomyosarcomas, desmoid-type fibromatosis and schwannoma [3]. The estimated incidence of GIST in the Netherlands was 12.7 per million inhabitants in 2003 [4]. Studies in other countries report incidences between 7.8 and 21.1/million [5–10]. Most studies were non-nationwide, doctor-driven cancer registry studies [11].

Primary treatment remains surgery and when non-resectable, imatinib has considerably improved prognosis of these patients [2, 12–15]. Response to imatinib and progression-free survival depend on mutational status [16, 17]. KIT is the most commonly mutated gene (76.2–83.6%), followed by PDGFRA (3.2–11.2%) [18, 19]. A significant subset of the 10–15% of GISTs that lack mutations in KIT or PDGFRA are associated with loss of function of the succinate dehydrogenase complex, the so-called SDH-deficient GIST, which has specific histological features [20–24].

The diagnosis of GIST is based on morphology and CD117 and/or DOG1 immunohistochemistry [2, 19, 20, 25, 26]. Mutational analysis is considered standard of care in the diagnostic work-up for GIST for the first time in the 2008 ESMO guidelines, and after 2010, confirmation by an expert pathologist is recommended. [2, 27, 28] These recommendations are incorporated in the Dutch guidelines [29].

In 2004, a nationwide survey was performed in the Netherlands to estimate the incidence of GIST in 1995 and 1998 to 2003 [4]. We repeated this study for the following ten years (2003–2012) during which the diagnosis GIST was well established. Our primary objective was to estimate the incidence of GIST and the classification into the different risk categories, the frequency of the various mutations, immunohistochemical markers and histological subtypes. The secondary aim was to compare the current daily practice of pathology reporting with the actual ESMO guidelines.

## Methods

### Patients

From the PALGA, the nationwide network and registry of histo- and cytopathology in the Netherlands [30], all excerpts were retrieved matching the following search criteria: GIST or metastasis of GIST OR ((malignant) leiomyoma (i.e. leiomyoma, leiomyosarcoma and leiomyoblastoma) AND gastrointestinal tract). A second search was performed with the following criteria (used earlier by Goettsch et al. [4]): (gastro-intestinal tract OR abdomen OR retroperitoneal OR abdominal wall) AND (liposarcoma OR desmoid-type fibromatosis OR solitary fibrous tumour OR schwannoma OR malignant peripheral nerve sheath tumour). The standardised excerpts contain encrypted patient identification, age at diagnosis, sex, the date of arrival of the pathology specimen, whether the analysis was done in a clinical centre active in GIST (defined below) and the conclusion of the pathology report. Patients with a first, incident GIST were included. AJV extracted the data, and uncertain pathology conclusions in the reports were discussed with HG and JVMGB. For uncertain cases, full pathology reports were retrieved. Because not all questions could be answered with the information in the excerpts, full pathology reports were retrieved for all patients with a primary resection for a GIST in 2011 or 2012.

A clinical centre active in GIST was defined as a centre with more than 15 new pathology diagnosis of GIST per year and a dedicated multidisciplinary sarcoma team. Five Dutch centres met these criteria: the Erasmus Medical Centre, Rotterdam, the Antoni van Leeuwenhoek Hospital, Amsterdam, the University Medical Centre Groningen, the Radboud University Medical Centre, Nijmegen, and the Leiden University Medical Center.

### Data collection

Data was collected on age at diagnosis, sex, year of diagnosis, localisation, tumour size, mitotic rate, immunohistochemical staining results (CD117, DOG-1, SDHB, desmin, smooth muscle actin and CD34), mutation analysis and surgical resection margins. Tumour size *and* mitotic rate were categorised into to the categories used in the various risk classifications, i.e. < 2, 2–5, 5–10, > 10 cm *and* 0–5, 6–10, > 10 mitoses per 50 HPF or 5 mm^2^, depending on what was reported.

### Risk stratification scores

For the analysis of the different risk stratification scores, patients were grouped according to the criteria of Fletcher et al. [31], Miettinen et al. [32], revised Miettinen/AFIP [33], Joensuu [34] and Gold nomogram [35]. Most risk classifications give a long-term indication of the risk of recurrence, but the Gold nomogram specifies the 2- and 5-year recurrence-free survival (RFS) after surgery. For comparison, the 5-year RFS was used. RFS rates were categorised to a low risk group (Gold nomogram 5-year RFS 90–100%), moderate risk group (75–90%) and high risk group (< 75%), which are comparable to percentages given in the revised Miettinen/AFIP criteria. Because it is not possible in the RFS calculation to have a RFS > 96%, no very low risk group was identified.

### Statistical analysis

The incidence rate of GIST was calculated per million inhabitants, also standardised for 5 year age groups and sex for the Dutch population of 2012 and standardised to the WHO and European (ESR) standard population [36, 37]. Time trends for incidence were either tested for significance with regression analysis or a Mantel-Haenszel *Χ*^2^ test for trend. Spearman’s rank correlation coefficient was used to test the correlation between the different risk classifications.

## Results

**Fig. 1:**
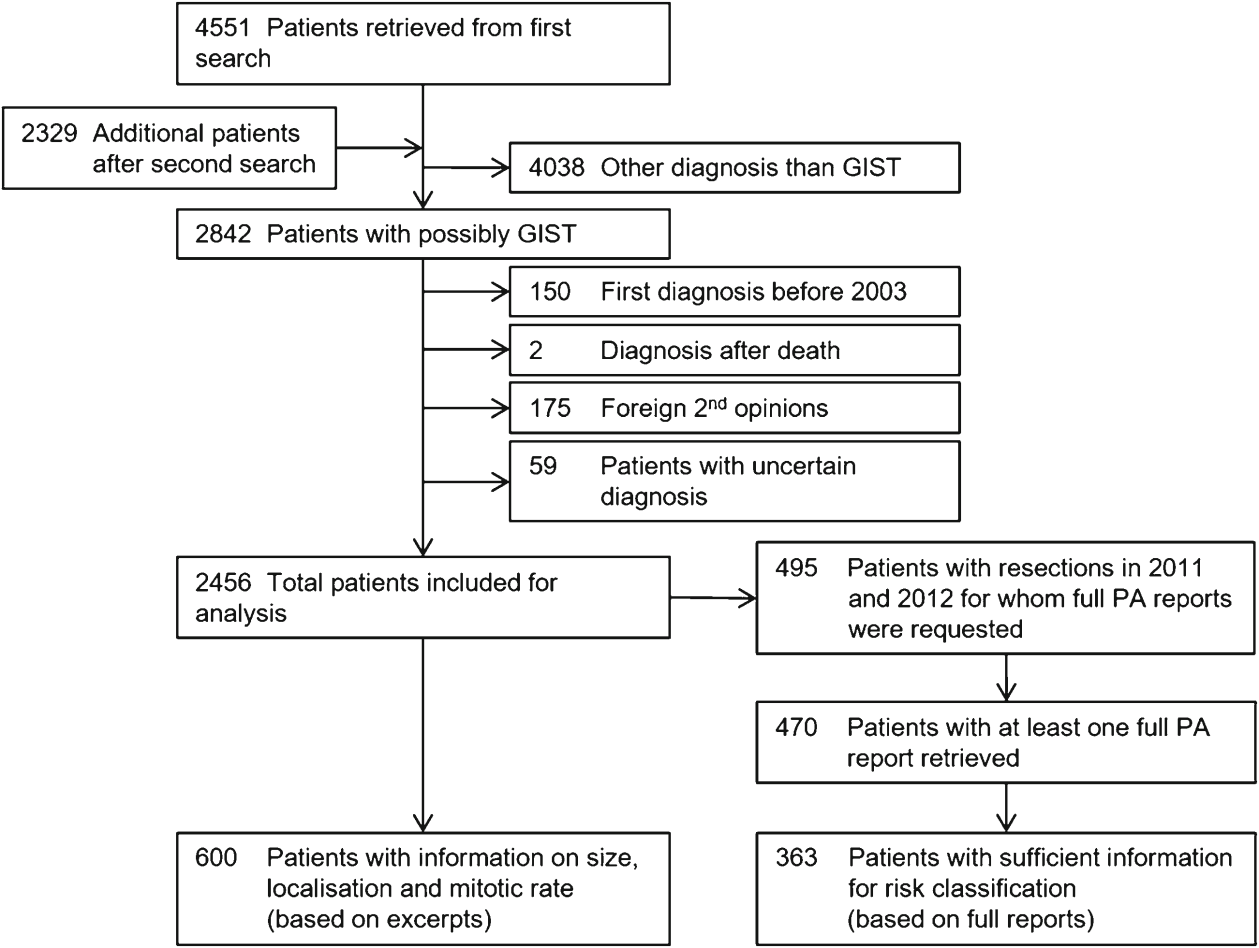
Diagram of inclusion and exclusion of patients

Figure 1 shows the search strategy and numbers of patients identified. In total, 2456 patients were included for incidence analysis and 489 patients were included for full pathology report analysis.

The mean age of patients was 65 years (SD 13), median 67 years (range 3–96) and 1307 (53.2%) patients were male (see also Supplementary Fig. 1). The localisation of the GISTs (patients with excerpts between 2003 and 2012) was the stomach in 59.8%, small intestine in 21.1%, rectum in 2.2%, colon in 1.6%, oesophagus in 0.6% and intra-abdominal not further specified in 11.0%. For the patients with full reports between 2011 and 2012, the localisation was stomach in 65.0%, small intestine in 26.8%, rectum in 3.1%, colon in 1.6%, oesophagus in 0.8% and intra-abdominal not further specified in 1.8%. The group with a small intestine GIST was further subdivided to duodenum in 6.1%, jejunum 5.1%, ileum 1.0% and not specified 14.5% (Supplementary Table 1).

Of the six patients < 21 years of age, four were female (3, 15, 18 and 20 years) and two were male (14 and 17 years). Localisations were the stomach (*n* = 4), colon (*n* = 1) and intra-abdominal not further specified (*n* = 1).

### Incidence rates

**Fig. 2:**
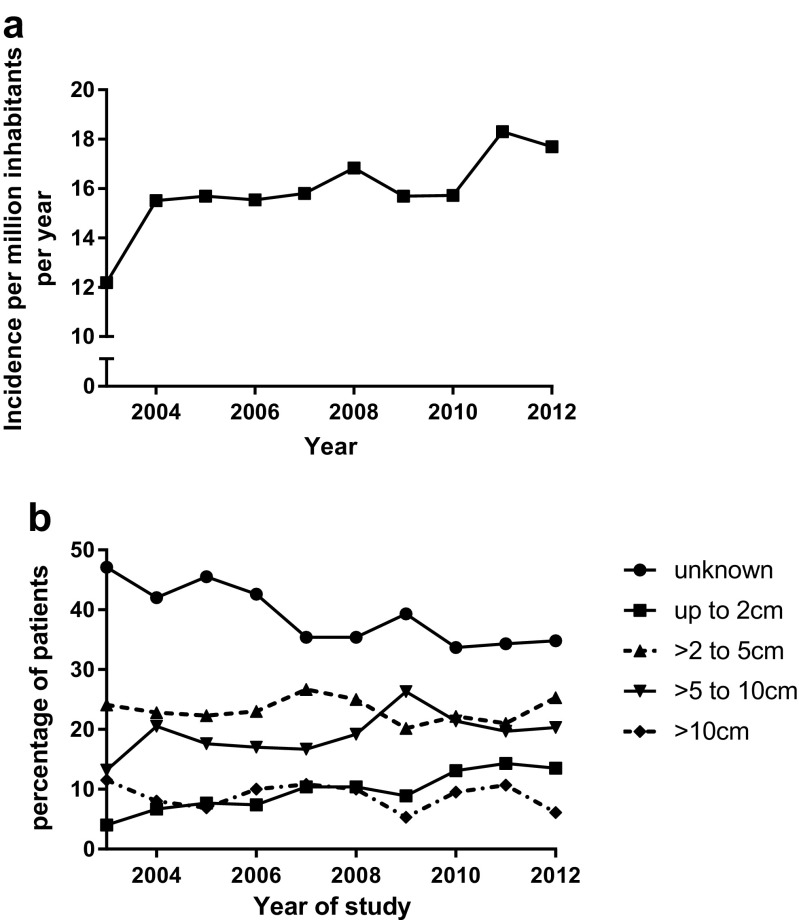
**a** Incidence of GIST standardised for the Dutch population of 2012. **b** Relative incidence of the four tumour diameter groups

The standardised incidence rate increased from 12.2 per million in 2003 to 17.7 in 2012 (*p* < 0.05). Age of peak incidence was 70–74 years with an incidence of 73.9 per million in 2012 for this age group. The incidence of GIST before the age of 21 was 0.13 per million per year (Table 1 and Fig. 2a).

**Table 1:** Incidence rates

Year	Absolute number of patients	Crude incidence rate, (patients per million inhabitants)	WHO age standardised incidence per million inhabitants	Standardised incidence (Dutch population 2012) per million inhabitants	European standardised rate per million inhabitants
2003	174	10.7	7.2	12.2	13.5
2004	224	13.8	9.3	15.5	17.2
2005	233	14.3	9.5	15.7	17.2
2006	230	14.1	8.9	15.5	17.2
2007	240	14.7	9.6	15.8	17.3
2008	260	15.8	10.1	16.8	18.5
2009	247	15.0	9.2	15.7	17.1
2010	252	15.2	9.6	15.7	17.1
2011	300	18.0	10.9	18.3	20.1
2012	296	17.7	10.8	17.7	19.4

During the 10-year study period, the proportion of tumours with a size <2 cm significantly increased (*p* < 0.0001) from 4.0 to 13.5% with at the same time a decrease in the proportion of patients for which tumour size is not reported from 47.1 to 34.8% (ns) with a stable absolute number (Fig. 2b).

### Histology

Detailed histological findings were only evaluated for patients with a full pathology report. Of the 429 patients (87.7% of patients with a full report) with known morphology, 81.6% had spindle cell morphology, 9.3% epithelioid subtype and 9.1% mixed epithelioid/spindle cell subtype. No differences in morphology were found for the specified localisations. For GIST patients < 21 years, histologic subtype was mixed morphology in two, epithelioid subtype in two and unknown in two patients.

Immunohistochemistry results were analysed for patients with full pathology reports. CD117 was reported in 89.4% of patients, and of these, 93.6% tested positive. For DOG1, 42.9% of patients were tested with a positive result in 98.6%. For additional results of immunohistochemistry, see Supplementary Table 2. For 49 patients (10.0%), no positive immunohistochemistry was reported for CD117 and/or DOG-1 in the full pathology reports or excerpts of the patients with a full pathology report. Only one was actually reported as being negative for both CD117 and DOG-1, and all others had at least one of both not reported. Resection margins were reported in 404 of 489 patients (82.6%) with a R0 resection in 84.9%, R1 in 11.6% and R2 in 3.5%.

### Risk classification

Full pathology reports were requested of all resections performed in 2011 and 2012. Of the 489 patients with at least one full pathology report, 414–444 patients had sufficient data for risk classification depending on the applied risk classification (because of different criteria not all classifications were able to classify the same patients). Although comparison of the incidence of risk categories is difficult because risk classifications differ in the number of patients eligible for risk stratification, both the Gold risk assessment and the Miettinen 2002 classification seem to allocate more patients to the highest risk group compared with the other risk classifications. All risk classifications had a significant and good to very good correlation (*p* < 0.001) with each other, with an *R* ranging from 0.808 (Gold vs Joensuu) to 0.957 (Miettinen 2002 vs Miettinen/AFIP) (Table 2 and Supplementary Table 3).

**Table 2:** Distribution of patients (with full reports) in the different risk classifications

Risk groups	2011–2012 (Full reports)	
	Absolute number of patients	Percentage of patients that could be stratified (not possible: percentage of all patients)
Fletcher 2002		
Very low risk	74	16.7
Low risk	137	30.9
Intermediate risk	109	24.5
High risk	124	27.9
Not possible	45	9.2
Miettinen 2002		
Probably benign	159	38.5
Uncertain or low malignant potential	97	23.5
Probably malignant	157	38.0
Not possible	76	15.5
Joensuu 2006		
Very low, if any malignant potential	66	16.2
Low malignant potential	191	46.8
Intermediate malignant potential	70	17.2
Probably malignant	81	19.9
Not possible	81	16.6
Miettinen 2006		
None	68	16.4
Very low risk	93	22.5
Low risk	101	24.4
Moderate risk	67	16.2
High risk	85	20.5
Not possible	75	15.3
Gold 2009 (chance of 5-year recurrence free survival)		
90–100% (low risk)	185	42.7
75–90% (moderate risk)	102	23.6
0–75% (high risk)	146	33.7
Not possible	56	11.5

Not all patients are present in every classification because they do not have all data essential for that classification

### Mutational status

Mutational status was reported in 461 of the 2456 patients (18.8%) based on excerpts and in 166 of 489 patients (33.9%) based on patients with full pathology reports. The presence of PDGFRA mutations is relatively high with a frequency of 16.3%. Supplementary Fig. 2 shows the distribution of mutated genes compared with age. The number of patients with mutational analysis performed increased during the years of study from 5.2% in 2003 to 29.4% in 2012 (*p* = 0.000).

The frequency of reported mutational analysis increased from low risk tumours (24.7%) to the high risk group (67.1%) (*p* = 0.000) (Supplementary Table 4 and Table 3).

**Table 3:** Mutation frequencies

Gene mutated	All patients after analysis of the excerpts 2003–2012		All patients with full pathology reports 2011 and 2012	
	Number of patients	Percentage of total known mutations (*n* = 461)^a^	Number of patients	Percentage of total known mutations (*n* = 166)^a^
KIT	322	69.8	112	67.5
Exon 9	30	9.3	11	9.8
Exon 11	261	81.1	97	86.6
Exon 13	8	2.5	2	1.8
Exon 17	1	0.3	1	0.9
Not reported	22	6.8	1	0.9
PDGFRA	64	13.9	27	16.3
Exon 12	5	7.8	1	3.7
Exon 14	3	4.7	3	11.1
Exon 18	46	71.9	22	81.4
Not reported	10	15.6	1	3.7
BRAF	1	0.2	1	0.6
SDHB deficiency	5	1.1	3	1.8
Neurofibromatosis	3	0.7	4	2.4
Wild-type, i.e. KIT and PDGFRA negative, in most patients no other mutation tested^b^	66	14.3	19	11.4

^a^For the exons: percentage of patients with a mutation in the specific gene

^b^Patients with a wild-type GIST were at least tested for mutations in KIT exons 9 and 11 and PDGFRA exons 12 and 18. Most of these patients were not tested for SDH deficiency or BRAF mutations

### Centres of diagnosis, resection and revision

Fifty two laboratories diagnosed GIST and 49 laboratories had at least one surgical resection specimen during the two years for which we requested full pathology reports. The pathology department of five GIST centres in the Netherlands diagnosed and revised more than 30 pathology resection specimens (> 15/year) of GIST in 2011 and 2012 (15 laboratories ≤ 5 specimens, 25 laboratories 6–20 specimens, 4 laboratories 20–30 specimens in these 2 years and 3 no specimen). If this cut-off of > 15 pathology specimens of GIST/year is used as definition of a GIST reference centre and with inclusion of all the regional soft tissue pathology panels, then for 13.2% of patients, the primary diagnosis was established in a GIST centre, surgery was done in 16.2% of the patients in a GIST centre and 35.9% of the patients were diagnosed or had a revision of their diagnosis within 3 months in a GIST reference centre. No significant increase was found in the number of pathology revisions over the years of study (2003 28.7%, 2012 41.2% of patients), although there seems to be an increasing trend in the number of reviews after the guidelines of 2010 (Supplementary Table 5).

It was also assessed whether the pathology specimens revised by a reference centre or specialised soft tissue pathology panel were high risk classified patients according to the Miettinen/AFIP criteria. Only 30.9% of the patients with a full pathology report with no risk for recurrence had a revision of the pathology diagnosis compared to 67.1% of the patients with a high risk (Supplementary Table 4). Of all patients with a resection and a revision in a reference centre, 61.2% had mutational analysis performed compared to 10.4% of all the other patients.

Last, we analysed whether high risk patients diagnosed in 2011 and 2012 had a mutation analysis. Of the patients diagnosed in a GIST reference centre, 92.3% had a mutation analysis, but only 16.7% of the patients diagnosed in one of the other centres.

## Discussion

The current study shows an increase in incidence of pathology proven GIST from 12.2 to 17.7 per million inhabitants between 2003 and 2012. This increase in incidence is also found in several other studies, like the SEER study (SEER database study, standardised to the 2000 US standard population, 2001: 5.5/million, 2011 7.8/million) [8], a Taiwanese study (Taiwanese Cancer Registry, standardised to the 2000 US standard population, 1998: 11.3/million, 2008: 19.7/million) [6] and last a study from Shanghai (Shanghai Cancer Registry, WHO standardised, 2004: 10.1/million, 2008: 14.5/million) [7]. None of these studies report a cause for this increase. Studies reporting incidences before 2000 report also an increase in incidence; however, this is caused by the introduction of CD117 immunohistochemistry to identify GIST. [10]

We can only hypothesise about the cause of the increase in The Netherlands. First, it could be an increased use of diagnostic procedures such as CT scans, gastroscopy and endoscopic ultrasound, which is supported by the increase in number of patients with a small tumour size. Another possible reason is an increased awareness of the diagnosis after the introduction of imatinib as effective treatment. The last possibility could be a real increase in the incidence; although this is a possibility, until now no causal factors or risk factors for the development of GIST are known.

The difference in crude incidence for 2003 in the Goettsch paper [4] and our paper (our data 174 patients vs. Goettsch 206 patients) could be explained by the revision of historical pathology specimens after 2003 or by improvements in patient identification by PALGA, resulting in less double counted patients for incidence analysis.

The incidence of 17.7 per million inhabitants is to the upper limit of reported incidences, although comparison is hampered by a lack of standardised incidence rates [5–10]. This high incidence rate is probably caused by one of the strengths of our study: the way PALGA registers diagnoses. PALGA is a fully automated archive of pathology reports, with 100% coverage of all Dutch pathology reports and registers also small and incidental GISTs not appearing in cancer registries. With the addition of the extensive search, the long study period and the inclusion of small and incidentally found GISTs, this study gives the best possible estimate of GIST incidence. Most of the earlier studies used cancer registries that use a health care provider notification system, which is probably biased as small and incidentally found GISTs are clinically less relevant as was shown in a recent study [11]. A Dutch Cancer Registry (DCR) study on rare cancers reported an incidence of 9 per million inhabitants for 2004–2008 compared to an incidence of 13.8 to 15.8 per million in our study [38]. The DCR is probably not registering small GISTs, explaining the difference.

The ESMO guideline of 2010 recommends to perform mutation analysis in all GISTs, because mutational status is related both to prognosis and efficacy of treatment. However, only a minority of patients in 2011 and 2012 (33.9%) had mutational status reported. [16, 17, 27] When considering high risk patients, mutational analysis was performed in 67.1% of patients [16, 17]. Because this study is based on pathology reports, exact reasons for not performing mutational analysis are not known. Almost all patients with a high risk GIST and a primary diagnosis or revision in a GIST centre had a mutational analysis (2011 and 2012 92.3%) compared with a much lower rate in the non-GIST centres (2011 and 2012 16.7%), explaining the rather low rate of mutational analysis performed in high risk patients and stressing the importance of referring patients to a GIST centre. The frequency of mutations was in line with that reported in a French study [5]. PDGFRa mutant GIST was slightly overrepresented, which may be explained by the imatinib resistance of PDGFRa-mutated GIST and therefore due to progression leading to an indication for mutation analysis [39]. The relative high percentage of patients which were characterised as wild-type could have technical reasons because most patients were only sequenced for KIT and PDGFRa mutations in the most common hotspots.

In the past, risk classification was not incorporated in the guidelines, and so, mitotic rate and size were often not reported in the conclusion. To get a better overview of the risk classifications, we requested full pathology reports for all patients with a resection in 2011 and 2012. Comparing the different risk classifications, it seems that the Gold and Miettinen 2002 criteria allocate more patients to the highest risk category compared with the other known risk stratifications, but comparison is difficult because these classifications do not include exactly the same patients in our analysis. For example, both the Joensuu and the Miettinen 2002 criteria do only provide stratification rules for gastric and intestinal tumours. Also, the number of risk groups differs between classifications. These factors hamper comparison of the different stratifications.

Since 2008 the ESMO guideline recommends mutation analysis for all GISTs and the 2010 guidelines recommends revision of pathology by an expert pathologist, we here show that in 2012 only 41.2% of patients had a revision of pathology within 3 months and only 29.4% of patients had mutational analysis performed. This was much better for high risk patients (based on the Miettinen/AFIP classification) with 67.1% for both mutational analysis and pathology review.

In conclusion, this is the second nationwide GIST incidence study ever performed in the Netherlands and follows the previous study in the Netherlands in 2003 [4]. It shows that the registered incidence of GIST has risen from 12.2 to 17.7 per million, which can be partly explained by an increase in the incidence of small GISTs. Both the Gold risk assessment and the Miettinen 2002 criteria seem to allocate more patients than the other commonly used risk classification systems to a high risk category. We found that the majority of pathology reports currently do not contain the recommended data of the ESMO guideline. So, the incidence of GISTs apparently increases, mainly due to the increase of small GIST and for these small GISTs, the guidelines are probably less well adhered to.

## Electronic supplementary material

ESM 1 (DOCX 54.4 kb)

ESM 2 (DOCX 263 kb)

ESM 3 (DOCX 13.5 kb)

ESM 4 (DOCX 12.7 kb)

ESM 5 (DOCX 13.4 kb)

ESM 6 (DOCX 12.2 kb)

ESM 7 (DOCX 12 kb)
