# Supplementary figures and images for: The incidence, mutational status, risk classification and referral pattern of gastro-intestinal stromal tumours in the Netherlands: a nationwide pathology registry (PALGA) study
Source: Virchows Arch. 2018 Jan 8;472(2):221–9. doi: 10.1007/s00428-017-2285-x (PMC5856869; doi:10.1007/s00428-017-2285-x)

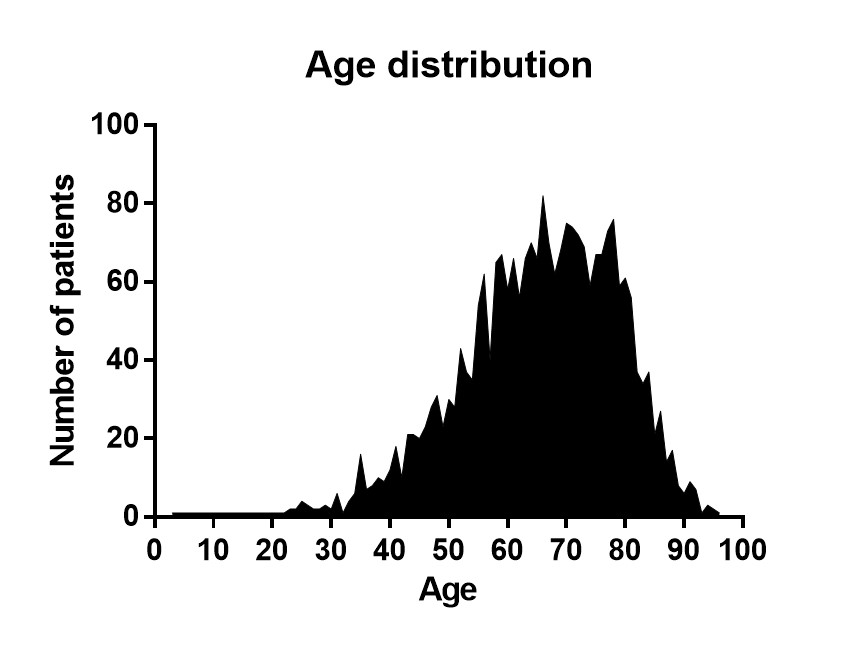


Supplementary figure 1: Age distribution

Supplement: Supplementary file 1 — (DOCX 54.4 kb) [file 428_2017_2285_MOESM1_ESM.docx]
