# Supplementary material for: The incidence, mutational status, risk classification and referral pattern of gastro-intestinal stromal tumours in the Netherlands: a nationwide pathology registry (PALGA) study
Source: Virchows Arch. 2018 Jan 8;472(2):221–9. doi: 10.1007/s00428-017-2285-x (PMC5856869; doi:10.1007/s00428-017-2285-x)
Supplement: Supplementary file 2 — (DOCX 263 kb) [file 428_2017_2285_MOESM2_ESM.docx]

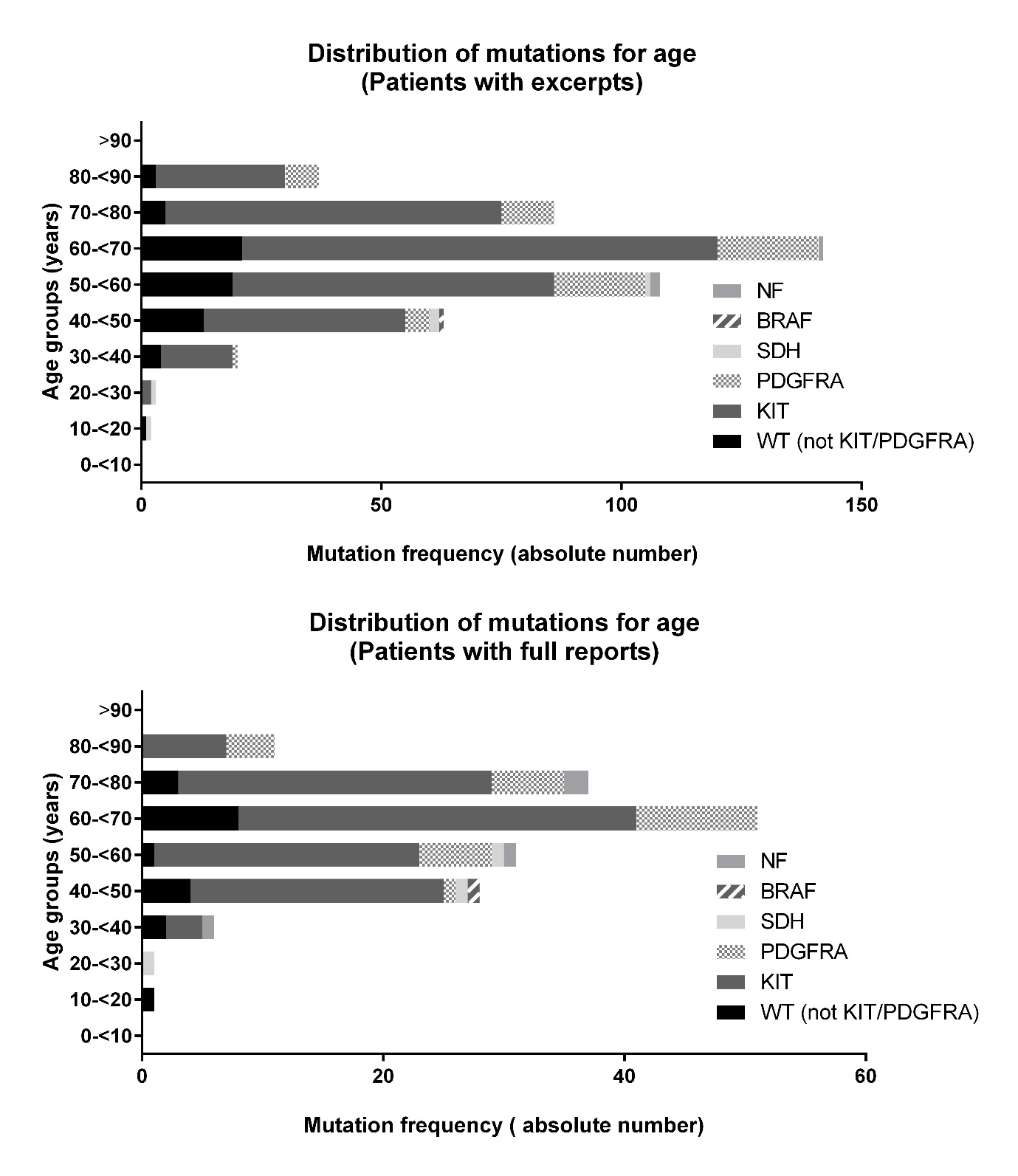


**Figure 2: Distribution of mutations for age**

Wild-type GIST patients were tested for mutations at least in KIT exon 9, 11 and PDGFRA exon 12 and 18. Most of these patients were not tested for SDH deficiency or BRAF mutations.
