# Supplementary material for: The incidence, mutational status, risk classification and referral pattern of gastro-intestinal stromal tumours in the Netherlands: a nationwide pathology registry (PALGA) study
Source: Virchows Arch. 2018 Jan 8;472(2):221–9. doi: 10.1007/s00428-017-2285-x (PMC5856869; doi:10.1007/s00428-017-2285-x)
Supplement: Supplementary file 3 — (DOCX 13.5 kb) [file 428_2017_2285_MOESM3_ESM.docx]

Supplementary table 1: Localisation of GIST

| **Localisation** | **Patients with excerpts**  **2003-2012**  (also containing the patients with a full pathology report 2011-2012) | | **Patients with full**  **pathology reports**  **2011-2012** | |
| --- | --- | --- | --- | --- |
|  | **Number** | **Percentage** | **Number** | **Percentage** |
| **Stomach** | 1469 | 59.8 % | 318 | 65.0% |
| **Small intestine** | 521 | 21.1 % | 131 | 26.8% |
| **Duodenum** | 89 | 3.6 % | 30 | 6.1% |
| **Jejunum** | 94 | 3.8 % | 25 | 5.1% |
| **Ileum** | 33 | 1.3 % | 5 | 1.0% |
| **Not specified** | 305 | 12.4 % | 71 | 14.5% |
| **Rectum** | 53 | 2.2 % | 15 | 3.1% |
| **Colon** | 39 | 1.6 % | 8 | 1.6% |
| **Oesophagus** | 14 | 0.6 % | 4 | 0.8% |
| **Liver, most probably metastases** | 46 | 1.9 % | 2 | 0.4% |
| **Pancreas** | 11 | 0.4 % | 1 | 0.2% |
| **Intra-abdominal, not further specified** | 270 | 11.0 % | 9 | 1.8% |
| **Other** | 25 | 1.0 % | 1 | 0.2% |
| **Unknown** | 8 | 0.3 % | 0 | 0.0% |
| **Total** | 2456 | 100.0 % | 489 | 100.0% |
