# Supplementary material for: The incidence, mutational status, risk classification and referral pattern of gastro-intestinal stromal tumours in the Netherlands: a nationwide pathology registry (PALGA) study
Source: Virchows Arch. 2018 Jan 8;472(2):221–9. doi: 10.1007/s00428-017-2285-x (PMC5856869; doi:10.1007/s00428-017-2285-x)
Supplement: Supplementary file 4 — (DOCX 12.7 kb) [file 428_2017_2285_MOESM4_ESM.docx]

Supplementary table 2: Results of immunohistochemistry

| **Marker** | **Full pathology reports** | |
| --- | --- | --- |
|  | **Percentage of patients in which it is reported** | **Patients with a positive result** |
| **CD117** | 89.4% | 93.6% |
| **DOG1** | 42.9% | 98.6% |
| **SDHB deficiency^1^** | 1.8% | 33.3% negative |
| **CD34** | 72.4% | 77.4% |
| **Desmin** | 60.7% | 0.7% |
| **Smooth muscle actin** | 51.7% | 19.4% |

^1^ Recently introduced and only of interest in KIT/PDGFRA wild-type GIST
