# Supplementary material for: The incidence, mutational status, risk classification and referral pattern of gastro-intestinal stromal tumours in the Netherlands: a nationwide pathology registry (PALGA) study
Source: Virchows Arch. 2018 Jan 8;472(2):221–9. doi: 10.1007/s00428-017-2285-x (PMC5856869; doi:10.1007/s00428-017-2285-x)
Supplement: Supplementary file 5 — (DOCX 13.4 kb) [file 428_2017_2285_MOESM5_ESM.docx]

**Supplementary table 3: Distribution of patients in the different risk classifications (all excerpts)**

| **Risk groups** | **2003-2012 (Excerpts)** | |
| --- | --- | --- |
|  | **Absolute number of patients** | **Percentage of patients that could be stratified**  **(not possible: percentage of all patients)** |
| **Fletcher 2002** | | |
| **Very low risk** | 89 | 9.9% |
| **Low risk** | 241 | 26.8% |
| **Intermediate risk** | 208 | 23.1% |
| **High risk** | 362 | 40.2% |
| **Not possible** | 1556 | 63.4% |
| **Miettinen 2002** | | |
| **Probably benign** | 252 | 30.4% |
| **Uncertain or low malignant potential** | 182 | 21.9% |
| **Probably malignant** | 396 | 47.7% |
| **Not possible** | 1626 | 66.2% |
| **Joensuu 2006** | | |
| **Very low, if any malignant potential** | 79 | 10.1% |
| **Low malignant potential** | 359 | 46.1% |
| **Intermediate malignant potential** | 136 | 17.5% |
| **Probably malignant** | 205 | 26.3% |
| **Not possible** | 1677 | 68.3% |
| **Miettinen 2006** | | |
| **None** | 82 | 10.8% |
| **Very low risk** | 173 | 22.9% |
| **Low risk** | 185 | 24.4% |
| **Moderate risk** | 133 | 17.6% |
| **High risk** | 184 | 24.3% |
| **Not possible** | 1699 | 69.2% |
| **Gold 2009 (chance of 5-year recurrence free survival)** | | |
| **90-100% (low risk)** | 285 | 35.6% |
| **75-90% (moderate risk)** | 190 | 23.8% |
| **0-75% (high risk)** | 325 | 40.6% |
| **Not possible** | 1656 | 67.4% |
