# Supplementary material for: The incidence, mutational status, risk classification and referral pattern of gastro-intestinal stromal tumours in the Netherlands: a nationwide pathology registry (PALGA) study
Source: Virchows Arch. 2018 Jan 8;472(2):221–9. doi: 10.1007/s00428-017-2285-x (PMC5856869; doi:10.1007/s00428-017-2285-x)
Supplement: Supplementary file 6 — (DOCX 12.2 kb) [file 428_2017_2285_MOESM6_ESM.docx]

**Supplementary table 4: Reference centre review and mutation analysis compared to Miettinen/AFIP risk group**

| Miettinen/AFIP risk group (N=) | | Percentage mutation analysis | Percentage reference centre review |
| --- | --- | --- | --- |
| None | (68) | 8.8 % | 30.9 % |
| Very low | (93) | 24.7 % | 32.3 % |
| Low | (101) | 29.7 % | 33.7 % |
| Moderate | (67) | 43.3 % | 38.8 % |
| High | (85) | 67.1 % | 67.1 % |
| Unknown | (75) | 36.0 % | 48.0 % |
