# Supplementary material for: The incidence, mutational status, risk classification and referral pattern of gastro-intestinal stromal tumours in the Netherlands: a nationwide pathology registry (PALGA) study
Source: Virchows Arch. 2018 Jan 8;472(2):221–9. doi: 10.1007/s00428-017-2285-x (PMC5856869; doi:10.1007/s00428-017-2285-x)
Supplement: Supplementary file 7 — (DOCX 12 kb) [file 428_2017_2285_MOESM7_ESM.docx]

**Supplementary table 5: reference centre review during years of study**

| Year of diagnosis | Reference centre review within 3 months after diagnosis |
| --- | --- |
| 2003 | 28.7 % |
| 2004 | 26.8 % |
| 2005 | 36.1 % |
| 2006 | 28.7 % |
| 2007 | 40.4 % |
| 2008 | 25.8 % |
| 2009 | 32.8 % |
| 2010 | 35.3 % |
| 2011 | 38.7 % |
| 2012 | 41.2 % |
